# Supplementary material for: HCN channels at the cell soma ensure the rapid electrical reactivity of fast-spiking interneurons in human neocortex
Source: PLoS Biol. 2023 Feb 6;21(2):e3002001. doi: 10.1371/journal.pbio.3002001 (PMC9934405; doi:10.1371/journal.pbio.3002001)
Supplement: S1 Table — Part I includes 72 fast-spiking cells studied for systematic sag amplitude in the whole-cell mode (evoked by hyperpolarizing steps to −90 mV from −70 mV). From left to right: experiment code, patient sex (M = male, F = female), patient age in years (yrs), resected neocortical tissue hemisphere (left or right), cortical area resected, patient primary diagnosis for surgery, sag amplitude (Ih sag, mV) measured during voltage steps from −70 mV to −90 mV, width of action potential escape current inward component (in ms) measured at the onset point of the fast depolarizing current [35], resting membrane potential (Em), firing accommodation index measured as the ratio of spike numbers within the 400–500 ms and 0–100 ms time windows during a 500-ms depolarizing pulse inducing high-frequency but not maximal firing [35,62], “accommodation Hz” indicates action potential firing frequency during the 0–100 ms time window of the depolarizing pulse used for accommodation index calculation; immunohistochemistry shows details of pv immunoreactivity (pv+ = pv-immunopositive in soma-dendrite or axon; nonconclusive means unsuccessful pv immunostaining, no recovery means unsuccessful cell visualization with biocytin. Part II summarizes the patient data for the resected tissues used in immunohistochemical studies of HCN1, HCN2, and Kv3.1 (Figs 2 and 3). Table shows tissue block identification code, patient data, and the immunochemical (IHC) study performed. S1 Data shows line analysis intensity values for all cells studied for HCN1 expression (8 patients) and HCN2 expression (6 patients). Part III summarizes the data from 13 fast-spiking human cells examined by both whole-cell and outside the out patch recordings and the corresponding patient data. Indicated as o-o patch cells 1 to 13 and studied with hyperpolarizing step to −90 mV from −60 mV. (DOCX) [file pbio.3002001.s002.docx]

| code | gender | age (yrs) | hemisphere | cortical area | diagnosis | sag (mV) | Axon current width (ms) | Em (mV) | Firing accomm index | Accom tested at Hz | IHC |
| --- | --- | --- | --- | --- | --- | --- | --- | --- | --- | --- | --- |
| cell h1 | F | 55 | left | temporal | tumor | 0.5 | 0.53 | -55 | 1 | 110 | PV+ |
| cell h2 | M | 68 | right | periventricularis | tumor | 1 | 0.47 | -55 | 0.9 | 100 | PV+ |
| cell h3 | F | 40 | right | frontal | anaplastic ependymoma | 1 | 0.7 | -74 | 0.8 | 200 | PV+ |
| cell h4 | F | 28 | right | temporal | hydrocephalus | 1 | 0.39 | -72.4 | 0.90625 | 320 | PV+ |
| cell h5 | F | 40 | right | frontal | anaplastic ependymoma | 1 | 0.49 | -71.9 | 1 | 90 | PV+ |
| cell h6 | F | 50 | left | occipital | astrocytoma | 3 | 0.54 | -68 | 0.857142857 | 140 | PV+ |
| cell h7 | F | 19 | right | frontal | subcortical neoplasia | 15 | 0.62 | -67.6 | 0.875 | 80 | PV+ |
| cell h8 | F | 54 | right | frontal | hydrocephalus | 2 | 0.69 | -63.8 | 0.625 | 80 | PV+ |
| cell h9 | F | 54 | right | frontal | aneurysm | 4 | 0.4 | -81.3 | 1.041666667 | 240 | PV+ |
| cell h10 | F | 68 | right | temporal | hydrocephalus | 0.5 | 0.4 | -75 | 0.95 | 200 | PV+ |
| cell h11 | F | 21 | left | temporal | tumor | 5 | 0.45 | -62.43 | 0.692307692 | 130 | PV+ |
| cell h12 | F | 42 | left | fronto-temporal | astrocytoma | 2 | 0.62 | -73.1 | 0.944444444 | 180 | PV+ |
| cell h13 | F | 32 | left | frontal | tumor | 2 | 0.41 | -69.6 | 0.842105263 | 190 | PV non concl. |
| cell h14 | F | 63 | right | temporal | tumor | 2 | 0.48 | -59.7 | NA | 150 | PV+ |
| cell h15 | F | 53 | left | temporal | tumor | 1 | 0.43 | -65 | 0.954545455 | 220 | PV+ |
| cell h16 | F | 30 | right | frontal | tumor | 0 | 0.59 | -61.2 | 0.875 | 80 | PV+ |
| cell h17 | F | 30 | right | frontal | tumor | 0 | 0.64 | -60 | 0.882352941 | 170 | PV+ |
| cell h18 | M | 20 | right | temporal | tumor | 1.5 | 0.57 | NA | 1 | 70 | PV+ |
| cell h19 | M | 43 | right | frontal | tumor | 14.5 | 0.53 | -55.4 | 0.857142857 | 70 | PV+ |
| cell h20 | M | 65 | right | temporal | tumor | 2 | 0.55 | NA | 0.714285714 | 70 | PV+ |
| cell h21 | M | 65 | right | temporal | tumor | 1 | 0.47 | NA | 0.857142857 | 70 | PV+ |
| cell h22 | M | 63 | right | frontal | tumor | 1 | 0.44 | -51.3 | 0.916666667 | 120 | PV+ |
| cell h23 | F | 55 | right | frontal | tumor | 4 | 0.67 | -57.7 | 1 | 110 | PV+ |
| cell h24 | F | 79 | right | parietal | hydrocephalus | 4 | 0.63 | -47.9 | 1 | 100 | PV+ |
| cell h25 | F | 66 | right | frontal | tumor | 1 | 0.63 | -55 | 1 | 90 | PV+ |
| cell h26 | M | 45 | right | temporal | trauma | 3.5 | 0.65 | -52.3 | 0.909090909 | 120 | PV+ |
| cell h27 | M | 68 | NA | periventricularis | tumor | 3 | 0.52 | -50.1 | 1 | 90 | PV+ |
| cell h28 | M | 33 | right | frontal | hydrocephalus | 5 | 0.59 | -66.45 | NA | 140 | PV+ |
| cell h29 | F | 72 | right | temporobasal | tumor | 7 | 0.61 | -65 | NA |  | PV+ |
| cell h30 | M | 47 | left | frontotemporal | tumor | 7 | 0.69 | -65 | 1 | 100 | PV+ |
| cell h31 | M | 70 | left | temporal | tumor | 3 | 0.66 | -67.4 | 0.916666667 | 120 | PV+ |
| cell h32 | M | 47 | right | temporal | tumor | 6 | 0.71 | -62 | NA |  | PV non concl. |
| cell h33 | F | 72 | left | temporal | tumor | 5 | 0.64 | -65 | 0.777777778 | 130 | PV+ |
| cell h34 | M | 66 | right | frontal | tumor | 0 | 0.55 | -58 | NA | 80 | PV non concl. |
| cell h35 | M | 66 | right | frontal | tumor | 5 | 0.66 | -55 | 0.8 | 60 | PV+ |
| cell h36 | F | 43 | left | parietal | hydrocephalus | 5 | 0.51 | -51.2 | 1 | 140 | PV non concl. |
| cell h37 | F | 35 | right | frontal | tumor | 4 | 0.68 | -49.8 | 0.875 | 90 | PV non concl. |
| cell h38 | M | 43 | right | frontal | tumor | 0 | 0.62 | -64.5 | NA | 120 | PV non concl. |
| cell h39 | F | 26 | left | parietal | tumor | 5 | 0.56 | -64.9 | NA |  | PV+ |
| cell h40 | F | 68 | right | temporal | hydrocephalus | 0 | 0.6 | -70 | 0.8 | 120 | PV+ |
| cell h41 | F | 68 | right | temporal | hydrocephalus | 6 | 0.62 | -65.9 | 0.833333333 | 120 | PV+ |
| cell h42 | F | 67 | right | occipital | subcortical neoplasia | 8 | 0.58 | -61.9 | 0.894736842 | 190 | PV+ |
| cell h43 | F | 54 | right | frontal | aneurysm | 5 | 0.44 | -45 | 1 | 170 | PV+ |
| cell h44 | F | 69 | right | occipital | subcortical neoplasia | 8 | 0.71 | -50.8 | 0.923076923 | 130 | PV+ |
| cell h45 | F | 63 | right | temporal | hydrocephalus | 0 | 0.7 | -50.2 | 1 | 150 | PV+ |
| cell h46 | F | 76 | right | frontal | hydrocephalus | 2 | 0.81 | -61.3 | 0.625 |  | PV+ |
| cell h47 | F | 54 | right | frontal | aneurysm | 5 | 0.56 | -56.2 | 0.8 | 120 | PV non concl. |
| cell h48 | M | 56 | left | temporal | aneurysm | 0.5 | 0.6 | -46 | 0.8 | 80 | PV non concl. |
| cell h49 | M | 56 | left | temporal | aneurysm | 3 | 0.71 | -51.9 | 0.9 | 80 | no recovery |
| cell h50 | M | 56 | left | temporal | aneurysm | 7 | 0.66 | -52.7 | 0.9 | 100 | no recovery |
| cell h51 | F | 39 | right | frontal | hydrocephalus | 0 | 0.59 | -51.75 | 0.947368421 | 190 | no recovery |
| cell h52 | M | 58 | right | temporal | tumor | 2 | 0.58 | -56.05 | NA | 100 | no recovery |
| cell h53 | M | 30 | left | frontal | tumor | 0 | 0.68 | -72.9 | 0.818181818 | 110 | PV non concl. |
| cell h54 | M | 30 | left | frontal | tumor | 2 | 0.57 | -53.95 | 0.909090909 | 110 | PV non concl. |
| cell h55 | F | 66 | right | frontal | tumor | 7 | 0.42 | -58.41 | 0.962962963 | 270 | PV non concl. |
| cell h56 | F | 57 | right | frontal | tumor | 9 | 0.41 | -69.9 | NA | 70 | PV+ |
| cell h57 | F | 57 | right | frontal | tumor | 8 | 0.58 | -58.3 | 0.85 | 200 | PV non concl. |
| cell h58 | F | 78 | left | frontal | hydrocephalus | 7 | 0.69 | -47.5 | NA | 40 | no recovery |
| cell h59 | M | 65 | left | ventral | tumor | 2 | 0.52 | -42.17 | NA | 40 | no recovery |
| cell h60 | M | 65 | left | ventral | tumor | 12 | 0.68 | -50 | 0.857142857 | 70 | no recovery |
| cell h61 | F | 47 | right | NA | colloid cyst tumor | 7 | 0.55 | -55.2 | 0.777777778 | 90 | no recovery |
| cell h62 | F | 47 | right | NA | colloid cyst tumor | 2.5 | 0.6 | -66.8 | NA | 110 | no recovery |
| cell h63 | F | 47 | right | NA | colloid cyst tumor | 4 | 0.45 | -65 | 1 | 240 | no recovery |
| cell h64 | F | 63 | right | temporal | tumor | 5 | 0.55 | -68.7 | NA | 30 | no recovery |
| cell h65 | M | 37 | NA | frontal | tumor | 1 | 0.61 | -61.9 | 0.823529412 | 170 | no recovery |
| cell h66 | M | 55 | NA | frontal | tumor | 0 | 0.42 | -74 | 0.935483871 | 310 | PV non concl. |
| cell h67 | M | 38 | right | temporal | anaplastic ependymoma | 3 | 0.57 | -60 | NA | NA | PV+ |
| cell h68 | F | 51 | right | frontal | hemorrhage | 0.5 | 0.52 | -65 | NA | 100 | PV non concl. |
| cell h69 | F | 21 | right | frontal | hydrocephalus | 4 | 0.6 | -60 | NA | 100 | no recovery |
| cell h70 | F | 51 | right | frontal | hemorrhage | 2 | 0.45 | -63 | 0.833333333 | 70 | PV non concl. |
| cell h71 | F | 21 | right | frontal | hydrocephalus | 0 | 0.55 | -61 | NA |  | PV+ |
| cell h72 | F | 82 | left | temporal | myoblastoma | 14 | 0.7 | -55 | 1 | 40 | no recovery |

**Part II**

| code | gender | age (yrs) | hemisphere | cortical area | Diagnosis | IHC |
| --- | --- | --- | --- | --- | --- | --- |
| 1 | male | 11 | left | temporal | hydrocephalus | HCN1 |
| 2 | female | 72 | right | temporal | tumor | HCN1 |
| 3 | female | 61 | right | frontal | tumor | HCN1 |
| 4 | female | 55 | left | temporal | tumor | HCN1 |
| 5 | female | 65 | right | parietal | aneurysm | HCN1 |
| 6 | female | 53 | left | temporal | tumor | HCN1 |
| 7 | female | 54 | right | temporal | tumor | HCN1 |
| 8 | male | 70 | left | temporal | tumor | HCN1 |
| 9 | female | 65 | right | frontal | colloid cyst | HCN2 |
| 10 | male | 38 | right | temporal | tumor | HCN2 |
| 11 | female | 61 | right | temporal | hydrocephalus | HCN2 |
| 12 | female | 65 | right | parietal | aneurysm | HCN2 |
| 13 | female | 54 | right | temporal | tumor | HCN2 |
| 14 | male | 38 | right | frontal | tumor | HCN2, Kv3.1 |

**Part III**

| code | gender | age (yrs) | hemisphere | cortical area | diagnosis | Axon current width (ms) | Em (mV) | Firing accomm index | Accom tested at Hz |
| --- | --- | --- | --- | --- | --- | --- | --- | --- | --- |
| o-o patch 1 | male | 70 | right | frontal | ventriculostomia | 0.75 | -62 | 1 | 90 |
| o-o patch 2 | female | 82 | left | temporal | glioblastoma | 0.76 | -60 | 1 | 80 |
| o-o patch 3 | male | 23 | right | occipital | hydrocephalus | 66 | -50 | 1 | 90 |
| o-o patch 4 | male | 23 | right | occipital | hydrocephalus | 43 | -59 | 1 | 80 |
| o-o patch 5 | male | 23 | right | occipital | hydrocephalus | 81 | -65 | 0.89 | 130 |
| o-o patch 6 | male | 20 | right | parietal | hydrocephalus | 0.75 | -68 | 1.13 | 80 |
| o-o patch 7 | male | 16 | left | parietal | hydrocephalus | 0.79 | -65 | 0.92 | 120 |
| o-o patch 8 | male | 16 | left | parietal | hydrocephalus | 0.54 | -60 | 0.92 | 130 |
| o-o patch 9 | male | 38 | right | temporal | tumor | 0.87 | -70 | 0.91 | 110 |
| o-o patch 10 | male | 38 | right | temporal | tumor | 0.71 | -64 | 1 | 110 |
| o-o patch 11 | male | 38 | right | temporal | tumor | 0.87 | -68 | 0.89 | 90 |
| o-o patch 12 | male | 47 | right | temporal | tumor | 0.85 | -60 | 0.89 | 90 |
| o-o patch 13 | male | 47 | right | temporal | tumor | 0.71 | -52 | 1 | 80 |
