# Supplementary material for: HCN channels at the cell soma ensure the rapid electrical reactivity of fast-spiking interneurons in human neocortex
Source: PLoS Biol. 2023 Feb 6;21(2):e3002001. doi: 10.1371/journal.pbio.3002001 (PMC9934405; doi:10.1371/journal.pbio.3002001)
Supplement: S2 Table — Columns from left: Current = Transmembrane current type and conductance (g, nS) in soma, dendrite, and axon. E (mV) = reversal potential of current; p = exponent of activation term. (DOCX) [file pbio.3002001.s003.docx]

| Current | *g_soma_* | *g_den_* | *g_ax_* | *E* | *p* | *V_m,1/2_* | *V_m,sl_* | *V_h,1/2_* | *V_h,sl_* | *τ_m,max_* | *τ_m,min_* | *V_tm,1/2_* | *V_tm,sl_* | *τ_h,max_* | *τ_h,min_* | *V_th,1/2_* | *V_th,sl_* |
| --- | --- | --- | --- | --- | --- | --- | --- | --- | --- | --- | --- | --- | --- | --- | --- | --- | --- |
|  | nS | nS | nS | mV |  | mV | mV | mV | mV | ms | ms | mV | mV | ms | ms | mV | mV |
| Na | 1100 |  | 3400 | 50 | 3 | -32 | 11 | -57 | -14 | 0.8 | 0.1 | -68 | 30 | 8 | 0.2 | -74 | 30 |
| HCN | 1.8 | 1.6 |  | -30 | 1 | -73 | -16 |  |  | 350 | 50 | -45 | 50 |  |  |  |  |
| K_d_ | 40 |  | 142 | -72 | 4 | -26 | 14 |  |  | 10 | 0.5 | -70 | 30 |  |  |  |  |
| K_ir_ | 1.2 | 0.9 |  | -72 | 1 | -88 | -12 |  |  | 10 | 1.0 | -45 | 35 |  |  |  |  |
| M | 0.6 |  | 0.5 | -72 | 1 | -35 | 18 |  |  | 1000 | 20 | -48 | 33 |  |  |  |  |
