# Supplementary material for: HCN channels at the cell soma ensure the rapid electrical reactivity of fast-spiking interneurons in human neocortex
Source: PLoS Biol. 2023 Feb 6;21(2):e3002001. doi: 10.1371/journal.pbio.3002001 (PMC9934405; doi:10.1371/journal.pbio.3002001)
Supplement: Attachment — Submitted filename: RESPONSE TO REVIEWERS.docx [file pbio.3002001.s004.docx]

Short summary for all Reviewers:

I am pleased to tell you that we have strengthened the study with extensive additional new experiments and analyses. We have added two completely new figures. For the Reviewers 1 and 3 request we now present stronger evidence for soma membrane HCN channel expression with specific antibody and STORM super-resolution immunofluorescence microscopy. Our new figure 2 presents STORM microscopy investigation of HCN1 localization in human and mouse interneurons. The results show compelling evidence for membrane-associated localisation of HCN protein at human but not at mouse cell somal membrane. To best of our knowledge, this is first time STORM super-resolution microscopy is utilized in analyses of human brain tissue samples in such detail.

For Reviewer 1 we have fully revised and strengthened the computational modelling section of the study. We have redesigned and run computational simulations with a more sophisticated model with new collaborator and co-author. We present these data in new figure 5 (and in a supplementary figure 2). With help of the advanced model we now demonstrate integrative effects of somatic HCN channels on human interneuron excitability and function.

For Reviewers 1 and 2, we analyse (with markedly increased data set) age, brain region variability, patient primary diagnosis and gender against the human cell data. We show statistically strong evidence that the age/brain region etc. variability fails to explain the strong HCN signal in human cells.

Finally, in the revised version we clarify our statements about HCN channels in human and mice. We emphasize that this functional feature has probably evolved in human cells to compensate their otherwise tardy somatic membrane potential kinetics - which inevitably arises from the low somal membrane leakiness. We conclude that by help of somatic HCN channels, the human interneurons can substantially accelerate their signal processing rate, which is a key component of many computational processes in the brain.

Point-to-point Response to the Reviewers:

Reviewer #1.

(1) There is a difference in the developmental stage between human and mouse samples. To minimize potential bias caused by the difference in the developmental stage, the authors should obtain additional recordings from adult and aging mice.

We apologise that we did not make it clear in the text that absence of somatic Ih sag potential, and absence of notable effect of HCN pharmacological blocker on somatic membrane potential or the excitability, are widely-characterized in fast-spiking interneurons in the mouse neocortex from adolescence to adulthood (Povysheva NV et al. 2008, 2013; Goldberg EM et al. 2011; Albertson AJ et al. 2017; Willems JGP et al. 2018; Yang SS et al. 2018; Jimenez-Sosa MS 2020). Those studies cover various neocortical regions, including the frontal cortex (which is area with largest sample size here in the human dataset) giving consistent results with only a negligible Ih sag or its entire absence in somatic recordings from fast-spiking interneurons. In fact, Ih sag only exists in mouse fast-spiking interneurons at early postnatal and early juvenile age, and it virtually disappears in adolescence. Absence of Ih sag in mouse fast-spiking cells remains from adolescence to adulthood (Goldberg EM et al. 2011; Yang SS et al. 2018).

Therefore, rather than reproducing the earlier findings on mouse, we have increased sample size of human neurons (from 42 to 71 cells) and performed Ih sag strength analysis against the human patient age. The analysis show that robust Ih sag is present in human fast-spiking cells through the entire adulthood from 20 year-old to 80 year-old patients. The human neuron dataset does not show correlation between Ih sag amplitude and human age. These new data are presented in the revised manuscript results -section and illustrated in new Figure 1D.

We hope that these new analyses, together with better explanation on what is already known in the literature about mouse cells, convinces the expert Reviewer that the observed difference in Ih sag strength between human and mouse cannot be explained by age bias.

(2) There is insufficient evidence to indicate that HCN channels are expressed in human pv+BC somata. Due to the electrical coupling between dendritic, somatic and axonal compartments, dendritic and axonal HCN channels, in essence, can influence the somatic membrane potential. In agreement with this idea, Figure 3Ai of this manuscript indicates that HCN1 signals are distributed to both the dendrite and soma of the labeled pv+BC.

Our reply to this concern comes in two parts.

First, we have performed new anatomical experiments for immunofluorescence signal localisation in human and mouse parvalbumin-immunopositive interneurons in the layer 2/3. We use dSTORM super-resolution microscopy (in collaboration with Dr Miklos Erdelyi at Department of Optics and Quantum Electronics) to localise immunofluorescent molecules in these cells. Our new experiments spatial resolution for immunofluorophore positioning is 64 nm x 64 nm bin size. Experiments in five human patient and in three mouse -tissue samples reveal that in human pv cells the immunoreaction for HCN1 in cell soma is heavily concentrated at the soma membrane. In contrast, in mouse pv cells HCN1 reaction in soma is weak and localised in somatic cytoplasm (we suggest that the cytoplasmic signal refers to HCN1 protein under transportation to distal areas, presumably to axon). As a reference to HCN1 localisation we study immunofluorescence signal for Kv3.1 potassium channel which is a well-characterised membrane-bound protein at pv cell soma membrane. In human cells. localisation of HCN1 and Kv3.1 in soma is very similar showing strong signal at the soma membrane. In contrast, in mouse only Kv3.1 signal is at soma membrane and HCN1 signal localises in soma cytoplasm. We think that these new anatomical experiments quite convincingly increase evidence that HCN1 is expressed at pv cell soma membrane in human fast-spiking interneurons.

Second, we strengthened electrophysiological evidence for somatic location HCN channels in human fast-spiking interneurons. For instance, we now include an experiment with outside-out nucleated macro-patch (Figure 1D) in human fast-spiking interneuron showing robust Ih-sag in somatic compartment of the cell pulled out from slice. We found that Ih sag amplitude is nearly similar when recorded in whole-cell configuration or in the isolated soma compartment.

We have also substantially increased n-number of fast-spiking cells in electrophysiological analysis studying effect of HCN-blocker, ZD7288, on cell resting membrane potential (Em) during somatic recording. Somatic Em level is a good parameter to reveal consistent or tonic somatic channel activity in somatic recording since direct-current conductances far from soma are unlikely to regulate somatic Em. Our data on 22 human fast-spiking cells show that somatic Em level is hyperpolarized several millivolts by ZD7288, whereas in mouse it is not.

We would like to also stress that we do not argue here against presence of HCN channels in fast-spiking interneurons dendrites or axon. We simply emphasise that these channels are present in cell soma (or very close to it) in human, and that in this subcellular localisation they regulate human cells in a way that HCN channels localised in dendrites or axon are unable to do.

(3) The authors used a single compartment model to simulate how HCN channels contribute to the EPSP-to-action potential coupling. In agreement with a previous study, the authors have shown that HCN channels alone produce an excitatory effect by increasing the absolute peak voltage of subthreshold EPSPs. In the presence of KCNQ channels, however, the net effect of HCN channel activation can be either excitatory or inhibitory as a function of EPSP amplitude. Furthermore, the co-activation of HCN and KCNQ channels is expected to reduce the membrane time constant to a larger degree than HCN channels alone. Since pv+BCs functionally express KCNQ channels, the authors should consider implementing a KCNQ conductance in their model.

This is an excellent point. We have completely redesigned the computational study in the manuscript. Computational simulation of real human fast-spiking interneuron is now using the single-cell model by Dr Attila Szucs, a specialist in computational neuron studies with expertise on HCN channel modelling in neurons (Szabó et al. PLoS Comput Biol. 2021 doi: 10.1371) (Rátkai et al. Sci Reports. 2021 doi: 10.1038).

The single compartment model used in the revised computational study

enables more detailed and realistic conductances by specified channels in the cells.

We simulate current-voltage relationship of neurons to hyperpolarising steps replicating the voltage responses measured in individual real cells (see new Figure 5 and the supplementary figure 2). Indeed, simulations of the conditions in the presence of ZD revealed additional somatic conductance components, activated by hyperpolarisation, in these cells. The current-voltage relationship indicates clear presence of Kir (GIRK) channel current in soma. Although we could not confirm M-current in the simulated fast-spiking cell, another simulated yet unidentified interneuron shows it. We now report these in the manuscript.

As a summary, we identified as far as the model allows us to, specific ‘other’ current components (Kir in fast-spiking interneuron; M-current in non-fast-spiking interneuron) in human interneuron soma and we report these in the manuscript. By help of our new simulation study with all the current components we can well replicate real cell function in control conditions and in the presence of ZD7288. Indeed, our simulations give realistic picture about ‘how turning somatic HCN current on and off’ changes the simulated neurons operation.

We have also re-written the manuscript discussion.

Minor

-The authors referred to 'HCN inactivation' (the second paragraph on page 5, the last paragraph on page 6, the legend of Figure 2A1 on page 17). HCN channels do not inactivate. Instead, HCN channels deactivate in response to membrane potential depolarizations.

We apologise the mistake. We have corrected this.

-Human pv+BCs were recorded from 'non-pathological neocortical tissue'. Since pathological conditions (for example, epilepsy) can change the expression of HCN channels in cortical neurons, the authors should provide a more detailed description of their criterion for selecting 'non-pathological neocortical tissue'.

We now provide a supplementary table (suppl. table 1) listing human cells included in the study with the primary neurological diagnosis resulting for patient’s surgery. As part of our research standard in this project we excluded tissue from patients with diagnosed epilepsy.

-The authors used the dynamic-clamp method to simulate excitatory synaptic inputs to pv+BCs. A dynamic-clamp system converts a defined conductance to currents based on the value of the membrane potential. Consequently, the output of a dynamic-clamp system is a current rather than a conductance (third line of the first paragraph on page 28).

This is now changed as suggested.

-How is the fast-spiking phenotype quantitatively defined in this manuscript?

We report parameters used for the identification in a supplementary table 1. For every human neuron included in this study we have confirmed either electrophysiological fast-spiking cell phenotype comprising (a) fast spike kinetics (defined as narrow 0.4ms to 0.7 ms width of axon inward current) and (b) negligible firing frequency adaptation (calculated from sustained firing elicited by 500 ms depolarising square-pulse current step), or/and positive immunohistochemical reaction of the cell for parvalbumin. The characterisation used here for fast-spiking cells was originally introduced in first Petilla conference review paper (Nat Rev Neurosci. 9:557-568, 2008), and we have used it in human, rat and mouse cells also in our several previous studies. In our recent study we showed with a comprehensive statistical analysis that electrophysiologically-only-defined human fast-spiking cells are not different from the cells with electrophysiological and immunohistochemical (pv+) identification (Szegedi et al. 2020).

-Is the difference between Em and Em-sag in Figure 2B2 significant?

Short answer: no, and yes. Yet, in the revised version this figure panel does not exist, and the results are reported in the manuscript text as follows:

‘Thus, the HCN deactivation-reactivation voltage step cycle applied at Em reveals contribution of HCN channels to somatic Em with a depolarizing effect significantly larger in human fast-spiking cells (P < 0.001, Mann–Whitney U-test), while Em values were not different (P = 0.09, Mann–Whitney U-test).’

- The color code in Figure 3A is confusing. In Figure 3Ai, the green signal represents pv, and the red signal represents HCN channels. By contrast, the green signal in Figures 3Aii and 3Aiii represents HCN channels. What the red signal in Figures 3Aii and Aiii represents is unclear to me because the color code is different from that of Figure 3Ai.

We have modified figures and color-coding in them.

- Although ZD7288 consistently reduced the leak conductance in Figure 3D, the sample size (five experiments) in Figure 3D is too small to determine whether this effect is statistically significant with a non-parametric test. Furthermore, the authors used the Mann-Whitney U-test (Wilcoxon rank-sum test) in this figure. To compare the leak conductance in control with that in ZD7288 in the same group of cells, the Wilcoxon signed-rank test is more appropriate.

We now test ZD effect with the Wilcoxon signed-rank test.

- A prediction is that ZD7288 can hyperpolarize the resting membrane potential of mouse pv+BCs, albeit to a smaller degree when compared with its effect on human cells. In sharp contrast, the authors found that ZD7288 had no effect on either the resting membrane potential (Figure 2B3) or the holding current (traces in Figure 3C1) in mouse pv+BCs. Thus, these results appear paradoxical when put together.

We have increased n-number in experiments testing ZD effect on Em. With larger dataset there is no effect by ZD either on Em in mice.

- 'Range from -95 mV to +40 mV': I think that '+40' should be '-40'.

We apologize the typo.

- Page 8, line 9, 'We voltage-clamped the cells at their Em (average in human of -59.0 mV": this value is inconsistent with the value in Figure 2A1 and B2 (-66.33 mV in human cells, Figure 2A1 legend).

We apologise for a typo in previous version of the manuscript.

- It is unclear how the effect of access resistance 'on the clamping potential error was corrected in nominal somatic potential reading' (page 27 in the Methods section). Please provide a detailed description of the correction procedure.

This description is now provided in the methods.

Reviewer #2.

General comments: Szegedi and co-workers report that putative PV+ basket cells from human tissue show a (possibly somatic) Ih current, whereas genetically-identified mouse PV-INs show lower values of Ih (largely identified in current-clamp recordings as voltage "sag"). They interpret this difference as a "human uniqueness" of human basket cells. However, it is known that rodent PV-INs show little Ih (the authors cite refs 33, 36-39, 43, 50 for this statement). Similarly, it has been reported before that PV-INs in macaque monkeys lack Ih (refs 33, 37, 50).

(1) Thus, the title and abstract of the paper over-emphasize the "specialization" of the human cells, and give only part of the story:

We agree that in previous version of the manuscript these statements were unclearly presented. We have rewritten the manuscript including title and abstract. Shortly, the general level message in our new fully-revised version is as follows:

-Instead of using expression ‘unique to human’, we present ‘this species-specificity’ of somatic HCN channel expression as an adaptation of human (and possibly monkey) neurons to compensate their ‘somatic tardiness’. Human cell soma tardiness, compared to mouse or rat, naturally emerges from large neuron size and soma surface area (hence, the somatic capacitance), resulting in slow passive membrane time constant (i.e slow responsiveness of cell soma membrane potential). We demonstrate this slow passive membrane tau in this study by blocking HCN channels with ZD.

-Slow tau in cell soma is disadvantageous to cell types which should operate fast and with fast responsiveness, like the cortical fast-spiking interneurons are (Hu H et al. 2014.). The slow passive membrane tau problem is particularly serious in human cells (and macaque monkey and maybe in other primates too), because human fast-spiking interneurons in addition have high input resistance due to low ion channel density (Szegedi et al. 2016, 2018; Eyal G et al. 2016).

-We propose that in human (and possibly in some other primates) fast-spiking neocortical interneurons have evolved with somatic HCN leak conductance to compensate their somatic tardiness. This idea we support here by demonstrating that the HCN conductance values measured in human cell somata indeed speed up their tardy passive somatic time constant (as revealed in ZD) and shorten action potential generation lag.

(2) The difference between human and rodent PV-INs is not merely a larger Ih in human PV-INs; rather, rodent PV-Ins seem to express different kind of leak conductances not present in the human cells (this was finally revealed in the discussion).

A major message in our revised manuscript is that it makes a difference to the fast-spiking cell function whether its somatic leak conductance is of ‘human style’ or ‘ mouse style’; in other words whether the soma leak conductance has HCN component or not. Shortly, in ‘the human style resolution’ the cells can increase their intrinsic excitability synergistically (our new figure 5) while speeding up somatic membrane tau.

Our completely redesigned modelling study in Figure 5 demonstrates this, and shows that somatic HCN channels are in many ways an ideal mechanism for compensation of slow passive membrane tau in human interneuron soma. HCN channels in soma make somatic tau faster (almost as fast as in some mouse cells) without reducing somatic excitability, since HCN current also significantly depolarises Em while speeding up somatic membrane potential kinetics. As a result, EPSPs are not only faster but they reach higher depolarising peak potential, when somatic HCN current is present. Figure 5 modelling data also demonstrates how this effect increases neurons input-output fidelity and shortens action potential lag.

In contrast, in mouse cells the fast membrane responsiveness (fast tau) is generated through K+ (and Cl-) leak (Földy C et al. Nature Neuroscience. 13: 1047-9, 2010). Yet, it is possible that the small mouse cells with smaller capacitance do not necessarily require extra HCN conductance at their soma membrane to reduce input resistance, and hence shorten the somatic membrane tau.

We discuss this aspect in a re-written discussion of the manuscript.

(3) Moreover, the effects of applying the Ih blocker ZD on the spike delay in human PV+ interneurons are quite modest, compared to the importance that this effect is given in the abstract.

We do not fully agree with the Reviewer here. We would like to emphasise that a typical ZD effect on spike delay in the experiments is roughly +30 % in cells with close-to-median size somatic Ih sag amplitude. We reproduce this result with the simulation study. This corresponds roughly to 1 millisecond in absolute time scale, simply because spike generation lag in fast-spiking interneurons is very short (3-4 ms). We show here that this ‘speed boosting’ is meaningful already in in single-cell level input-output operations (new figure 4, and figure 5), and we claim that the effect is strongly magnified in the neuronal network level operations.

(4) After having read the abstract, a non-specialist reader would assume that the membrane time constant of the "specialized" human PV-INs is faster than the ones in rodent PV-INs (which lack Ih). One is then surprised to find in the Results section that the leak conductance of rodent PV-INs is actually bigger than the one of human PV+ basket cells.

We apologise and we agree that our presentation was not clear in previous version of the manuscript. We have completely rewritten abstract, large parts of results, and the discussion. In the revised manuscript the topic is more clearly presented.

Minor

- Have the authors attempted to correlate their measured cellular parameters with patient age, and/or human brain area under study?

In the revised version of manuscript we test the human fast-spiking interneuron Ih sag strength against patient age, gender, and cortical region as well as hemisphere. In addition we study its potential correlation with patient primary diagnosis for surgery. We found that none of these parameters explain occurrence of robust Ih sag in human fast-spiking neurons. These data and statistical tests are reported in results of revised manuscript text and they are illustrated in a new figure 1 panels D1-3.

- Moreover, the authors should show that PV-INs in the "somatosensory" and "frontal" areas have similar properties.

In 42 mouse cells there were 21 in somatosensory cortex and 21 in frontal cortex. Sag values (0.00 mV, 0.00 to 1.5 mV) (0.00, 0.00 to 0.2 mV) were not different between the two groups (P = 0.238, Mann-Whitney U-test). We now report this in methods -section.

- Regarding the human tissue, it should be stated how many patients were donors for this study, their exact ages (not just a wide age range), sex; the diagnosis of each patient, and the resected brain area localization. Even if "overlaying" "non-pathological" tissue was used as stated by the authors in the Methods, it is important to know the exact brain pathology for each human sample studied here. Each recorded cell should then be linked (in a Suppl. Table or similar) to the data of the individual human sample (age, sex, brain area, diagnosis).

We provide this information now in detail in a supplementary table 1. In addition, this information is summarised in the manuscript text and in the Figure 1 panels D1-3 with statistical tests. The table lists the human fast-spiking cells (n = 71), number of patients, their exact ages, gender, their primary diagnosis for surgery, resected cortical area and hemisphere. In addition, the table shows Ih sag amplitude, and relevant parameters for their identification as fast-spiking pv interneuron (i.e. action current width, action potential firing accommodation, immunohistochemical reactivity for pv) as established in the Petilla conference review for interneuron classification (Nat Rev Neurosci. 9:557-568, 2008).

- In Figure 1, most human PV+ basket cells seem to have a sag between 0 - 5 mV; there are only 2 outliers with a much larger sag of ~ 13 - 15 mV. Correspondingly, the authors should show example traces in Figure 1A1 and 1B1 of more representative recordings (the examples have quite large sags of ~ 10 - 12 mV).

We show now example traces covering entire range of Ih sag amplitude strength in human and mouse fast-spiking cells.

- For ease of reading, please add the calibration values next to the scale bars in the Figures.

All figures have now calibrations values with the bars.

Reviewer #3.

(1) Sag Measures: This study claims that there is a leak conductance in the soma of PV basket cells in humans due to HCN Channels that is absent in their mouse counterparts.

-Sag measured at the soma is larger compared to the mouse but the range /variability in the mouse data set is very high.

For the revised manuscript we have increased n-number for both human (from 29 to 71 cells) and mouse (from 32 to 42 cells) data. Larger datasets allow more secure definition of their median and quartiles and confirm significant difference between the human and mouse cell data, although a range of the data values did not change.

-Further the estimation demonstrated is using the rebound sag measure and not the hyperpolarizing voltage Ih sag. I think it is necessary that you plot the standardly used Ih Sag ratio as (Vpeak - Vsteady-state)/Vpeak X 100%, Vpeak is the maximal hyperpolarized voltage and Vsteady-state is the steady-state voltage in response to a given hyperpolarizing current step relative to membrane holding potential (Kupferman et al., 2014) and plot the sag range as a function of the current injection step (Narayanan, Johnston, 2007)?

We agree that the data analysis and presentation for Ih sag amplitude were not conventional in the previous manuscript version hence we have changed it. In our new Figure 1, as well as in results section of the manuscript text, we now report Ih sag amplitude as ‘Vpeak – Vsteady-state’ measured at constant ‘Vpeak-value’ (-90 mV). Note that in al cells the hyperpolarising step to Vpeak (-90 mV) was delivered at -70 mV (Figure C1). In addition we show Ih sag amplitude in same cells by a voltage step to -70 mV peak potential at -50 mV (Figure 1C2). We think that this experimental design with standard (-20 mV) amplitude steps applied at two different membrane potentials (-50 mV and -70 mV) enables confident and straight-forward comparison of somatic Ih sag strength between individual neurons.

(2) It has been reported that HCN expression is also axonal in PV fast spiking neurons in rodents (Roth 2020; Aponte 2006). This is not discussed or addressed in the manuscript.

We agree that axonal HCN expression is common in pv interneurons for instance in the hippocampal dentate gyrus as those two papers (Roth et al. 2020; Aponte et al. 2006) and e.g. Elgueta et al. 2015 also show. We also mention now axonal HCN of fast-spiking cells here in our manuscript on neocortical pv cells, although these two (hippocampal formation and neocortex) are distinct brain areas which often exhibit differences in neuronal level.

-Please provide experimental evidence with co-staining with axonal markers and methods comparable to Aponte 2006 - (antidromic stimulation of axons) to show the presence or absence of HCN in human PV neuron axons.

In our opinion the question on axonal HCN expression and Ih axonal operation in human versus mouse neocortical pv interneurons is quite clearly out of scope of this manuscript. Axonal (presynaptic terminal vs. axon hillock) operation of HCN channels is an important and exciting topic, which deserved its own study; When expressed in axon, HCN channels are responsible for very different kind of modulation of cell function than what the same channels do in soma. In this manuscript our research focus is in somatic operation of the interneurons, and we would like to be cautious about commenting on axonal HCN in human neocortical pv interneurons.

(3) The major finding that there is a 'large' difference between human and mouse has been over emphasized.

We would like reply to this argument in two ways.

First, our substantially enlarged dataset (particularly in the human where we saw large Ih sag amplitude variation) consolidates the finding that Ih sag is indeed quantitatively much larger in human than in mouse fast-spiking cells. In addition, in human cells the Ih sag range is very large which means that in a notable subpopulation (e.g. upper quartile) of all fast-spiking cells, the difference between human and mouse is already pronounced (comparison of upper quartile roughly 5.5 mV in human vs. less than and 1 mV in mouse). Importantly, almost 70% of mouse fast-spiking cells completely lack a detectable Ih sag, whereas in human cells less than 10% of neurons do not show Ih sag at all.

Second, these are simply numerical differences, but we have made better effort to demonstrate what these Ih sag values (and hence somatic conductance behind their generation) really mean to basic function of a fast-spiking cell. Our new Figure 5, based on a sophisticated single-cell model by our collaborator Dr Attila Szucs, demonstrates how ‘turning on and switching off’ realistic somatic HCN conductance in human cells significantly change the neuron resting potential, time constant, EPSP depolarising peak potential, and consequently the spiking fidelity at Em, and a time delay in somatic input-output transformation. Shortly, our experimental data and the modelling results (Fig. 5C-E) reveal that a median-strength HCN activity causes clearly noticeable changes in cell function. Furthermore, modelling as well as our recorded data show that significant changes are observed in human cell function with much smaller than median-strength HCN.

we claim that the observed somatic HCN levels really make significant difference in the neurons basic operation. However, we also agree that in previous version of the manuscript this conclusion was not well presented and we did not select wording wisely.

-functional evidence. For example: Page 9 Regarding the effect of ZD on membrane Tau 'Together, these results indicate that HCN-channel blocker affects somatic passive membrane time constant more pronouncedly in human than in mouse pv+BCs.". Further, it is more pronounced in human but not by much. Thus, the difference is inflated and it is important to show data comparison of mouse vs human. It is more pronounced in humans but it is not mentioned/discussed why when there isn't much HCN in mouse, the effect on mouse is still comparable.

In the previous manuscript version we had a small sample size and, importantly, most human cells (5 cells) in the sample (8 cells) had Ih sag below the human population Ih sag median value. Correspondingly, in mouse cell sample 4 of 7 cells were in the upper quartile or above the median (and showed Ih sag). Therefore, we argue that although the results were statistically different between human and mouse, the cells did not well represent typical cells (with Ih sag close to a population median value). Yet, in individual cells with clear Ih sag the ZD effect on tau was obvious and clear, as we demonstrated in a cross-correlogram of sag amplitude vs. ZD effect on tau (Coefficient = 0.905, P > 0.001, n = 15 cells comprising both human and mouse).

The bias was obviously due to small sample size.

Can we measure tau in all our our 22 human cells? And in the mouse cells with ZD?

Yes, we have completed the analysis with 22 cells and added two new cells which increases dataset in human to 24 fast-spiking cells. In addition, the mouse dataset has increased to 12 pv cells. The data show that in human cells the membrane tau prolongs from 7.92 ms (median) to 11.38 ms (median). This corresponds to +43 % increase in membrane tau. Recordings in mouse show 5.76 ms (median) in baseline control conditions and 5.62 ms (median) in the presence of ZD. Results of the new data are now reported results-section.

-Also, in Page 10: "However, the effect of ZD on the EPSP time-to-peak was found to be larger in human compared to mouse pv+BCs (P = 0.049, Student's t-test; Shapiro-Wilk normality P = 0.34), increasing the baseline-normalized value to 1.18 ± 0.14 in human and to 1.07 ± 0.06 in mouse pv+BCs." Again same comment as before. The mouse effect isn't much different. P=0.049 - so barely meeting significance criteria.

This analysis suffered from having 3 out of 9 human cells with very small Ih sag diluting the overall result in human. We also conclude that EPSPs time-to-peak is less clearly-defined parameter to measure than e.g. membrane tau or action potential lag which can be measured with high accuracy. EPSP time-to-peak measurements are more susceptible to noise (variability in measuring ttp time point) than the other two parameters. Therefore, we think it is more confident to measure ZD effect by using membrane tau and spike lag, and although the EPSP ttp measurements are in line with our conclusion their evidence is weaker as a more noisy parameter. We therefore use EPSP as a measure in the revised version only in modelling data and the experimental measurements focus on membrane tau and spike lag.

(4) Figures need to be better annotated and mouse data has to be consistently presented side by side with the human data in the same format and analysis.

We have changed all images so that their presentation is now clearer and they are more logical to follow. We hope our changes here are in line with what the Reviewer expects.

Minor

- Please provide example traces from mouse in Fig 1

We now provide example traces for both human and mouse. In new Figure 1 the sample traces illustrate whole range of Ih sag amplitude responses observed in human and mouse populations.

- Please provide rebound calculation for the mouse data

In the revised manuscript we changed Ih sag presentation style, hence Ih resag data became redundant and repetitive, and are not shown.

- "We therefore examined the vertical position of pv+BCs soma in L2/3 to assess whether the voltage sag strength differed between cells localized in the superficial or deep L2/3 (Fig. 1C2)." The rationale for examining the sag correlation with soma depth is unclear and a better explanation to motivate this analysis is warranted.

This panel in the previous manuscript Figure 1 version was removed because the illustrations was seen redundant given that there is no correlation between Ih sag amplitude and cell soma location laminar depth.

Rationale for the correlation comes from an earlier observation that human and mouse pyramidal cells in layer 2/3 exhibit larger Ih sag in deep in L3 compared to PCs with soma in superficial L2/3 (Kalmbach et al. 2018).

-Page 8 "Thus, ZD reduced Gleak to 0.84 (average, range 0.57 to 1.04) from baseline control." Not to but 'by'

We have rewritten the paragraph.

-Please explain how you measured the Gleak conductance.

Gleak conductance measurement is explained now in methods section.

-Page 18: Figure legend "Voltage-clamping pv+BC at Em (−65 mV throughout the experiment) was associated with a moderate (+12 pA) shift of holding current level in ZD." This is different from what was stated in the main text.

We apologise this unclear expression in the text. We now explain this (i.e. the fact that ZD wash-in was associated with small shift in voltage-clamp holding current level) in less confusing way stating that ‘*Trace holding-current levels in figure are aligned for clear comparison of step amplitude change by ZD.*’

-Fig 4. A2. 0.008 not shown here human control vs in ZD, and the same for mouse

As mentioned above the dataset for membrane time constant was elaborated substantially and the data with statistical tests are now presented in the manuscript results section.
