# Supplementary material for: HCN channels at the cell soma ensure the rapid electrical reactivity of fast-spiking interneurons in human neocortex
Source: PLoS Biol. 2023 Feb 6;21(2):e3002001. doi: 10.1371/journal.pbio.3002001 (PMC9934405; doi:10.1371/journal.pbio.3002001)
Supplement: Attachment — Submitted filename: Response to Reviewers 25 Nov 22.docx [file pbio.3002001.s006.docx]

Our point-by-point responses to the Reviewers’ comments:

Reviewer #1: In comparison to the previous version of the manuscript, the current version has been significantly improved. Specifically, the authors have conducted additional experiments to support that HCN channels are expressed in human parvalbumin-expression basket cell (PV+-BC) somata. Furthermore, they have performed analyses to demonstrate that the targeting of HCN channels to human PV+-BC somata is independent of a range of different factors. Collectively, these results suggest that somatic HCN channels may be a widespread feature of human cortical PV+-BCs. However, the current manuscript still has several issues.

Major

(1) In nucleated patches from human PV+-BCs, a hyperpolarizing current pulse evokes a sag in the voltage response (Figure 1D). The authors use this result as the electrophysiological evidence to support the idea of somatic expression of HCN channels in human PV+-BCs, but this experiment has a weakness. Although activation of HCN channels can create a sag at the beginning of long-lasting hyperpolarizations, HCN channels are not the only mechanism that can give rise to the sag response. In essence, the sag can also be produced by the closing of slow and non-inactivating K+ channels, for example, KCNQ channels. Consequently, additional pharmacological experiments with ZD7288 are required to confirm that HCN channels can account for the sag response in nucleated patches from human PV+-BCs.

Reply: We thank the Reviewer for these suggestions. In fact, our electrophysiological experiments revealed slight spike frequency adaptation in human basket cells under excitation with positive square-pulse current steps, suggesting the activation of intrinsic slow K^+^ currents, particularly the M-current. We therefore included a moderate strength M-current in our model neuron (described in detail below) to mimic this firing frequency adaptation and assess its effects on voltage sag, rebound potential, and other physiological features.

To determine the electrophysiological effects of the M-current on voltage sag, we first simplified our model by removing the HCN and K_ir_ -currents, leaving only the spike generating conductances (Na_T_ and K_d_) and M-current. However, we found that an extremely small voltage sag (<1 mV) was actually produced under this condition, and this small voltage sag required depolarization to ≥50 mV. This is in contrast to the h current (HCN channel)-mediated voltage sag, which increases approximately linearly in amplitude with hyperpolarized membrane potentials. We have attached a figure (below) showing simulated responses in the presence of M-current only. The voltage sag generated by the M-current under these conditions occurs at membrane potentials that are much more positive than needed to induce the voltage sag in a biological neuron (sag is seen in biological neuron at Vm negative to −50 mV, generally from −70 to −90 mV). Further, the approximately linear increase in voltage sag in response to incremental hyperpolarizing current steps close to and beyond the potassium reversal potential (−90 to −95 mV) observed in biological human neurons indicates that the mechanism is unlikely attributable to the activation of a K^+^ current (M- or Kir-type). Based on these considerations, we conclude that M-current activation is not the major source of the voltage sag observed in biological human neurons over a wide range of membrane potentials in this study.

In addition, we simulated these responses in the presence of an inward rectifying K^+^ current (Kir) without including HCN and M-currents. Under this condition, only membrane potential hyperpolarization beyond the K^+^ reversal potential (−90 to −95 mV) produced a voltage sag, and this response was smaller and much faster than the real voltage sag observed in human basket cells in this study. The second part of the figure below presents the simulated responses under this condition. The sag amplitude measured at −90 mV provides a good estimate of h current strength (HCN channel activity) in biological neurons because of the small contributions of Kir and M-currents close to the K^+^ reversal potential (−90 to −95 mV).

|  | *Depolarizing voltage sag appearing in the I–V responses of model neurons with artificially enhanced strong M and Kir currents (in the absence of HCN channels). Families of voltage traces are shown during somatic current injection. Three selected traces from both models are magnified on the right. The membrane resistance and voltage sag are also shown as functions of injected current. Arrows in simulated traces indicate “sag” responses generated in the presence of nonphysiologically large M and Kir currents.* |
| --- | --- |

Finally, our outside-out patch recordings (see new Figure 4) revealed that the voltage sag in biological neurons exhibits a similar voltage dependence in whole-cell and somatic membrane outside-out patch configurations. Although we did not perform ZD7288 wash-in experiments because of the short recording duration achieved in the outside-out configuration, we argue that the present data on sag voltage dependence and retention of sag amplitude following the formation of outside-out patches provide strong evidence for mediation by HCN channels.

(2) Examples in Figure 2 qualitatively suggest that HCN channel expression pattern in human PV+-BCs is different from that of mouse interneurons. However, a quantitative analysis to compare the density of HCN1 immunosignal in human PV+-BC somata with its mouse counterpart is lacking.

Reply: We agree with the Reviewer that quantitative analysis of HCN immunoreactivity is needed. We have performed extensive additional immunofluorescence analysis based on confocal microscopy. In addition to HCN1, we have also examined the immunoreactivity of HCN2 in both human and mouse pv cells. These results are presented in the new Figure 2 and further demonstrate the perisomatic localization of HCN channels.

(3) The authors have described that HCN channels can accelerate the conversion of EPSPs to action potentials (APs) by reducing the membrane time constant (end of the first paragraph on page 10) and by depolarizing the resting membrane potential (page 14). However, there is one additional factor that has not been mentioned. The deactivation of HCN channels during an EPSP speeds up the EPSP repolarization (Magee JC, 1998, J Neuroscience), and this active property can help to restrict AP initiation to the early phase of EPSPs. This mechanism should be included in the discussion.

Reply: This mechanism is now also mentioned in (and highlighted in last paragraph of) the revised Discussion.

(4) My previous comment about the interplay between h- and M-currents during the EPSP-to-AP conversion of human PV+-BCs has not been sufficiently addressed. The minimum, which the authors should do, is to cite the relevant article and discuss this. KCNQ2, Q3 and Q5, the molecular correlates of M-current in neurons. This current activated between −60 and −55 mV, had a voltage-dependent activation time constant of 208 ± 12 ms at −20 mV, a deactivation time constant of 165 ± 24 ms at −50 mV. M-current is present in neurons, is activated at resting membrane potential and that it is physiologically important in regulating excitability by maintaining cells at negative voltages. (George MS et al., 2009, Nature Neuroscience), the authors have shown that HCN channels alone produce an excitatory effect by increasing the absolute peak voltage of subthreshold EPSPs. In the presence of KCNQ channels, however, the net effect of HCN channel activation can be either excitatory or inhibitory as a function of EPSP amplitude (George MS et al., 2009, Nature Neuroscience). Furthermore, the co-activation of HCN and KCNQ channels is expected to reduce the membrane time constant to a larger degree than HCN channels alone. Since pv+BCs functionally express KCNQ channels (Soh H et al., 2018, eLife), the authors should consider implementing a KCNQ conductance in their model.

Reply: Although pv-positive basket cells express KCNQ conductance, it appears that the somatic M-current is small in both human and mouse fast-spiking cells. The interneurons examined in this study exhibited slight firing frequency adaptation (see the firing frequency adaptation index in Supplementary table 1), which is often attributed to somatic M-current.

A closer analysis of the human pv cell I–V relationship revealed robust Kir and h current (HCN) but little M-current at the soma (see our new Figure 6). We thank the referee, however, for reminding us of the importance of M-current in modulating excitability; however, these effects are mostly substantial during sustained depolarization, which we did not examine in this study. Nonetheless, we included a realistic M-current in the neuron model simulations in which all basic experimental results were verified.

Furthermore, I do not think that supplementary figure 2 can help us to address this issue because EPSP-to-AP conversion has not been analyzed in this figure.

Reply: The supplementary image has now been removed from the manuscript.

Minor

(1) My previous comment 'The authors used the dynamic-clamp method to simulate excitatory synaptic inputs to PV+-BCs. A dynamic-clamp system converts a defined conductance to currents based on the value of the membrane potential. Consequently, the output of a dynamic-clamp system is a current rather than a conductance' has not been sufficiently addressed (last paragraph on page 9, Figure 4 legend on page 19 of the current manuscript). In a dynamic-clamp experiment, a signal applied to the input of the dynamic-clamp system is treated as a conductance instead of an EPSC.

Reply: We have revised the wording and highlighted relevant sentences in the results section and in Figure 5 legend.

Furthermore, siemens cannot be used as the unit to describe the magnitude of EPSCs (Figure 4B2legend).

Reply: We apologize for this mistake. It has been corrected in the figure legends.

(2) There is a mismatch between color codes in Figures 1A1 and 1B2. In Figure 1A1.  The biocytin signal is represented by red color, and the parvalbumin signal is represented by green color. In Figure 1B2, the color code is the opposite.

Reply: These colors reflect genuine fluorophore emission colors. Different conjugated fluorophores were used in the visualization of human and mouse cells and their pv signals. We have included the fluorophore used in each labeling experiment.

(3) When describing conductance and capacitance values of the model (page 25), please normalize the values with the membrane area of the model. This facilitates the comparison of the parameters in this paper with the result from a previous study, which shows that human cortical pyramidal neurons display unique passive membrane properties in comparison to their rodent counterparts (Eyal G. et al., 2016, eLife). Similarly, the magnitude of active conductances in the model should be described by using conductance density (Supplementary Table 2).

Reply: We have entirely reconstructed the model, and simulation results are shown in the new Figure 6, whereas details of capacitance density and axon longitudinal resistance density are described in the revised Methods section.

(4) Please describe which currents 'D' and 'A' in Supplementary table 2 represent. Are these D-type and A-type K+ currents? Why did the authors choose different reversal potential values for different types of K+ current (Supplementary table 2)?

Reply: We have provided the detailed descriptions of Kd, Kir, M, HCN, and Na+ currents in the Methods section. We apologize that two different reversal potential values were listed in the supplementary table. This error has been rectified.

Reviewer #2: Summary:

The authors, through electrophysiological recordings as well as immunohistochemical staining of parvalbumin positive interneurons (PVIs), show that human PVIs express more HCN channels in their somatic cellular membrane than the same cell type in the mouse cortex. The authors further show that in human PVIs HCN channels decrease the membrane time constant and thus facilitate faster action potential output in response to EPSP input close to AP threshold. These experimental results are corroborated by a computational single cell model with parameters based on one of the recorded cells, in which modification of HCN conductance produces similar effects as observed in the electrophysiological measurements.

In general, the study provides convincing evidence for a difference in HCN channel expression between human and mouse cortical PVIs. The resulting effects on membrane time constant and input-output delay are not unexpected but have interesting implications for the precision of inhibition in cortical circuits. However, some technical issues concerning the relation between sag measurements and underlying Ih conductances remain to be addressed.

Major Comments:

1. In figure 1, the authors show a wide range of sag amplitudes in the human PV interneurons. It would be interesting to discuss if this range is correlated with other parameters (pv level, cell size, baseline input resistance, dendritic/axonal morphology, etc.) since there are multiple sub-classifications of PV interneurons in the cortex.

Reply: We found no significant correlation of sag amplitude with input resistance, time constant, or any patient demographic parameter. However, we agree that an association with morphological parameters would be valuable for interneuron classification; therefore, we will continue to test for correlations with larger sample sizes.

2. One of the key difficulties in comparing sag values between different cell types is to determine whether any differences are due to differences in intrinsic Ih properties, such as peak conductance, steady-state voltage-dependent gating, or Ih kinetics, versus differences in extrinsic factors, such as membrane potential, input resistance of the cell, or the cell's membrane time constant. All of these can act as confounding factors. Although the authors attempt to control for some of these factors, there are some concerns. For example in Fig 1E, p5, 2nd paragraph, the authors compare sag in humans with a hyperpolarizing step to -91 mV with sag in mouse using a voltage step to -85 mV. This 6 mV difference can likely lead to a several-fold larger Ih conductance increase in human vs mouse, depending on the mid-point voltage of Ih activation.

Reply: We have accurately measured mouse cell sag values in steps to −90.1 mV; IQR, −89.3 to −91.0 mV. In addition, we increased the number of examined cells to 60 and updated Figure 1 accordingly.

The nicest was the authors have of controlling for such effects is through the outside-out patch data they show in Fig 1D. Unfortunately this is only shown for human cells and even there the number of experiments and mean date are not provided.

Reply: We have performed additional outside-out patch recordings from human fast-spiking cells, as identified using the parameters listed in Supplementary table 1. Results and analysis are presented (and highlighted) in the new Figure 4. We also performed experiments in mouse cells, although few had a measurable somatic sag amplitude even in whole-cell mode. Nonetheless, we obtained adequate data for neuron analysis.

As another means of assessing Ih in a manner that will be less prone to influence of extrinsic factors, the authors could measure Ih under voltage clamp from the data of Fig 3, subtracting the current in the presence of ZD from the current in the absence of ZD.

Reply: We calculated gHCN (leak conductance) in whole-cell voltage-clamp dataset to define the ZD-sensitive leak when the cell was held in its resting potential (baseline minus drug condition, see Figure 4). Although we measured the sag potential in current clamp mode, we believe that the outside-out soma membrane experiments provide compelling evidence that the sag amplitude dependence of membrane potential is due to somatic HCN channel activation.

3. The logic of the experiment of Fig 4b,c examining the role of Ih in action potential firing is rather unclear. I would have thought the primary goal would be to ask how spike firing is altered without the presence of Ih, without altering other parameters that are indirectly affected by Ih. In contrast, the authors artificially manipulate the resting potential to keep it constant and decrease the size of the EPSC to compensate for the change in input resistance due to loss of Ih. Although this might be of interest to examine why loss of Ih may alter spike firing, it does not provide an accurate view of how loss of Ih may affect cell firing. The authors should therefore repeat the experiments with ZD7288 but keeping the same amplitude EPSC as in control and allowing the resting potential to hyperpolarize to its appropriate level.

Reply: We have rewritten the Results section describing the dynamic clamp experiments to more clearly explain that our aim was to assess the effects of active HCN channels on membrane potential kinetics alone (especially the time constant). We explained that this experiment is crucial because it provides quantitative evidence that the change in tau alone can affect the cell input–output behaviour. In addition, we have substantially revised the modeling section (Figure 6) to include simulations with concomitant changes in Em, input resistance, and membrane potential kinetics (as would occur in a real neuron).

4. As ZD7288 may exert off target effects on sodium channels, the authors should also determine effects of threshold voltage and rheobase, both from a fixed holding potential (eg -70 mV) and from the actual resting potential measured with or without drug.

Reply: We apologize but we did not record an exact rheobase or determine firing threshold at the two membrane potentials used for most experiments. In the experiments presented in Figure 4, we focused adjusting the protocol so that EPSP–spike responses in the presence of ZD7288 were comparable to those at baseline.

5. Discussion paragraph 1: "In fact, these channels are necessary to reduce the EPSP-spike delay to roughly that of mouse basket cells." - This is a strong statement, given that figure 4C1 shows that even after ZD7288 the delay in mouse cells seems much shorter. The authors should tone down this conclusion.

Reply: We have extensively rewritten the Discussion section, including this particular sentence. The revised sentence is as follows (also highlighted in the text): “*In fact, these channels ensure that human cell* *EPSP–spike transformation reaches nearly the same speed as that in mouse pv neurons. Thus, in human fast-spiking neurons, perisomatic HCN channels appear to act as a mechanism ensuring rapid and reliable EPSP–spike coupling*.”

6. Discussion paragraph 2: "Further, the absence of other leak conductances may reduce the energy expenditure required to maintain transmembrane ionic gradients." - I am not quite clear on how another leak conductance could facilitate processing speed as alluded to in this paragraph. In fact, the effects attributed to HCN channels in this study might best be approximated by less leak current in the soma, which would result in an overall higher input resistance and faster time constant as well.

Reply: We have removed any speculation about differences in energy expenditure among adaptations to enhance EPSP–spike coupling speed.

Therefore, the claim that "somatic HCN channels in human neurons can be seen as an evolutionary adaptation that facilitates otherwise compromised signaling speed in circuits" may be a little simplistic.

Reply: The wording has been changed as follows (new text is also highlighted in the Discussion): *“**Somatic HCN channels in human pv neurons can be regarded as an evolutionary adaptation that compensates for inherently slow passive membrane kinetics, which is inadequate for fast-spiking cells.”*

Minor comments:

Abstract:

Page 2:
"… precisely timed inhibitory outputs that regulate within neural networks." - missing word (or just regulate neural networks)

Reply: We have corrected it as follows: “...regulate neural networks.”

"… jitter in the input-output function arise mainly during the transformation of excitatory
postsynaptic potential (EPSP) into action potential output." - should EPSP be plural (EPSPs)?

Reply: This has been revised to “EPSPs.”

"Of the four mouse cells; however, only showed a significant increase in delay" - word missing

Reply: This error (and misuse of the semicolon) has been corrected.

"Notably, we found a strong correlation between ZD7288-evoked lengthening of the spike delay and the somal membrane time constant" - somatic membrane time constant

Reply: The term “somal” has been revised to “somatic” throughout the manuscript.

Page 11:
"These so-call basket cells" - so-called

Reply: This error has been corrected.

Page 12:
"Somatic HCN channel activity is a common characteristic of these neurons in human but …" – humans

Reply: This error has been corrected.

"Despite being called fast-spiking neurons, basket cells in human neocortex actually exhibit…" - in the human neocortex

Reply: This sentence has been revised as follows: *“Indeed, basket cells in the human neocortex exhibit sluggish membrane potential kinetics at the resting potential when HCN channels are blockedt”*

Reviewer #3:

The paper by Szegedi et al. examines the effects of HCN channels on input-output transformation in fast spiking, GABAergic interneurons in the human brain. To address this question, the authors use electrophysiology, superresolution imaging, and modeling. The main findings are:
-        Fast-spiking parvalbumin-expressing interneurons show a high density of HCN channels in the perisomatic region in humans.
-        Somatic HCN channels enhance input-output fidelity, accelerate somatic membrane potential kinetics, and shorten the delay of action potential generation.
-        In human interneurons, somatic leak conductance is low and Ih conductance is high, whereas in mice the situation is opposite.

Based on these results, the authors conclude that the high perisomatic density of HCN channels in fast-spiking parvalbumin-expressing interneurons in human neocortex contribute to the fast signaling properties of these neurons. Overall, this is a nice paper. As very little information is available about GABAergic interneurons in humans, the manuscript could provide an important contribution to the scientific literature. However, it is also clear that major revision is required before the manuscript can be published in Plos Biology, including both additional experiments and major revision of the text. Given that this is a revised manuscript, I was surprised that parts of the paper were quite preliminary.

Major points:

1. The immunocytochemical analysis is preliminary and needs to be extended. First, the immunosignals shown in figure 2 need to be quantified, in terms of both number of labeled cells and labeling intensity.

Reply: We have conducted extensive quantitative analyses of HCN1 and HCN2 immunofluorescence signals in both human and mouse pv cells (new Figure 2). Briefly, we performed systematic line analysis of immunofluorescence intensity for both signals as described in the new Figure 2 and revised Methods section. Individual pv-expressing cells were analyzed using a radial six-line configuration centered on the soma and extending to the extracellular space. Fluorescence intensity (in arbitrary units) was normalized and averaged for each cell. In total, 2340 measurement lines from 390 pv cells were analyzed, which demonstrated higher HCN1 and HCN2 channel densities at the human cell soma membrane than those at the intra- or extracellular sites. Moreover, high-resolution analyses localized these channels to the membrane zone (the outer edge of the cell within which pv immunoexpression rapidly declines). In contrast, mice expressed these channels within the somatic cytoplasm. We hope that these new findings satisfy the Reviewer.

Second, the authors focus on HCN1, which is only one out of four potential subunits of HCN channels.

Reply: In the revised manuscript, we included new experiments analyzing the immunohistochemical staining patterns of HCN1 and HCN2 channel isoforms using both conventional confocal microscopy (Figure 2) and super-resolution dSTORM imaging (Figure 3).

HCN2 is also highly expressed  in parvalbumin-positive, fast-spiking interneurons (Aponte et al., 2006, J. Physiol.), and some hippocampal interneurons are also immunopositive for HCN3 (Notomi and Shigemoto, 2004, JCN).

Reply: Indeed, Aponte et al. revealed that HCN1 and HCN2 are expressed at equal levels in pv interneurons and suggested that they form heterodimers. In addition, human transcriptome analysis indicated that HCN1 is the predominant subunit in supragranular layer neocortical pv cells. According to Aponte et al. 2006, no HCN3 or HCN4 is expressed in pv interneurons.

Therefore, we argue that HCN1 and HCN2 subunits are the most logical choises for imaging studies examining whether HCN channels are preferentially localized at the perisomatic membrane.

Finally, the authors describe the expression of Ih as a black and white difference between rodents and humans. However, the data are more consistent with a quantitative difference. The statements needs to be attenuated.

Reply: We have extensively rewritten the manuscript (including the title) to better describe the similarities and differences between human and rodent basket cells as well as the contributions of Ih to their respective electrophysiological properties.

2. The authors interpret the findings as the difference in the somatic HCN channel density between human and mouse interneurons. However, differences in morphological properties are insufficiently considered. In the extreme case, it may be possible that the conductance densities in the different compartments were the same, and only the different morphological properties would explain the observations (see Mainen and Sejnowski, 1996, Nature, for the marked - indirect - effects of morphology on firing pattern).

Reply: We thank the reviewer for raising this issue. However, we argue that our new data strengthen the original conclusion that the sag amplitude difference between human and mouse mainly arises from HCN channel density at pv cell somatic membrane. We have presented evidence for differences in HCN1 and HCN1 channel densities using high-resolution fluorescence immunohistochemistry as well as outside-out patch-clamp experiments. We initially recorded data from a number of mouse and human fast-spiking interneurons in whole-cell configuration and then in outside-out patch mode under live DIC-IR camera imaging to confirm that patches in both configurations were at the soma. Switching from the whole-cell to outside-out configuration eliminated only a fraction of the relatively large sag potential in human cells, whereas in mouse cells, the sag amplitude was small in both configurations. We agree that anatomical features impact electrophysiological properties and we added citatio to Mainen and Sejnowski, 1996 (Nature) in the manuscript; however, using two independent methodologies (high-resolution immunofluorescence and outside-out somatic patches), we demonstrated that human basket cells express a large amount of HCN channles at the somatic plasma membrane while mouse basket cells do not.

Cable properties of fast-spiking parvalbumin-expressing interneurons in humans should be used to test this hypothesis.

Reply: We agree with the reviewer that primary dendritic localization of HCN channels does influence somatic voltage sag as demonstrated also by our outside-out ecperiments and neuron simulation. To address the effects of cable properties, we built a 3-compartment model for the simulation of human pv basket cell function, which includes a primary dendrite and axon with biologically realistic cable parameters (please see our new Figure 6). In the new simulations, the proportion of gHCN with dendritic localization was estimated based on the change observed between whole-cell and subsequent outside-out patch recordings (Figure 4). This change suggests that approximately one-third of the sag amplitude measured at the soma under normal conditions in whole-cell recording can be explained by dendritic HCN channels.

3. The cable modeling of the human parvalbumin-expressing interneurons needs to be improved. If the authors want to make the point that - somatic - Ih channels are important, they need to perform simulations with realistic morphology including dendrites, somata, and axon.

Reply: Based on the reviewer’s suggestion, we have refined our model by including the axon and dendrite compartments with realistic cable properties. The new model includes five voltage-activated currents, which are distributed across the axon, soma, and dendritic compartments. The upgraded model not only reproduces the dependence of membrane resistance, time constant, voltage sag, and rebound firing properties on gHCN but also exhibits moderate spike frequency adaptation as observed in human neurons. Additionally, the shape of the action potentials is reproduced in a better manner.

The upgraded model allows us to separately investigate the effects of perisomatic and more distally distributed HCN channels on intrinsic excitability and EPSP–spike coupling. Our previous findings regarding the enhancement of EPSP–spike transformation via perisomatic HCN channels in human neurons were confirmed using an upgraded model, which included a primary dendrite and axon with cable properties. The model results are shown in the revised Figure 6. The upgraded model cell closely mimics our dynamic clamp data on biological neurons with realistic EPSCs (as measured in soma), EPSPs, and EPSP–spike coupling.

Furthermore, it is not quite clear what exactly the authors did in their simulations. In the text on page 10, the authors state that they used a single-compartment model. In contrast, in the methods section on page 25, the authors mention that the model was derived from a compartmental model neuron including soma and initial axon segment. Furthermore, which program was used for solving the differential equations? Finally, what was the size of the time steps and the accuracy of the simulations?

Reply: The Results and Methods sections have been extensively revised. Simulations were conducted using a single-neuron model with three compartments as described. The program used for the simulations was written by A. Szűcs in Delphi language (Szücs et al. Eur. J. Neurosci. 2017; Hernáth et al. Sci. Reports 2019; Szabó et al. PloS Comp. Biol. 2021). The software uses Heun’s method of integration and a fixed time step of 1 μs to integrate the differential equations. Voltage-dependent currents can be distributed in a nonuniform manner across cable compartments, and the program also allows the simulation of virtual electrophysiological experiments, somatic current stimulation, and chemical synaptic or autaptic inputs targeting the dendritic or perisomatic regions. Our original model was a single compartment neuron, which was also developed in this computational environment and was refined by carefully tuning the cable parameters. We tested several methods of integration, including adaptive time steps and the 4-th order Runge–Kutta method, but the 1-μs fixed time step was found to be the most appropriate and efficient method for our cable modeling. This is because shorter time steps did not result in any visible difference in the model output. The inserted figure illustrates human neuron simulation mimicking a real cell membrane response to potential steps using modelling software.

4. The measurement of "membrane time constant" is not entirely correct. In a compartmental model, the membrane time constant is the - slowest - time constant in a multi-exponential waveform. If the authors want to maintain their analysis, they should denote the membrane time constant as "apparent membrane time constant".

Reply: This has been revised as advised by the reviewer.

5. The authors claim that ZD 7288 application causes a hyperpolarization of resting membrane potential in human, but not in mouse interneurons (p. 6, top). This is surprising, because ZD 7288 causes the robust hyperpolarization in interneurons in rats (Aponte et al, 2006). Furthermore, the authors should correct misleading statements in the introduction section (page 3, bottom, "small effect of HCN channel blockers on resting membrane potential").

Reply: The reviewer is correct but we want to emphasize that rodent pv cells appear to exhibit somatic sag (and ZD7288 effect on Em) in “evolutionarily old” brain structures, such as hippocampus and medial septum (Varga V et al. J Physiol 586, 3893-3915, 2008), but not in neocortex (whereas human pv cells show Ih-dependent sag in neocortex as well). Aponte et al. (2006) examined rat hippocampal pv cells. Studies on rodent neocortical pv (or fast-spiking interneuros) show small effect of HCN channel blocker on the cell soma including the resting membrane potential (Albertson et al. 2017; Goldberg et al. 2010; Yang et al. 2018). We have emphasized that our findings apply to human versus rodent neocortical cells throughout the revised manuscript. We have rewritten Introduction and removed that original sentense and new formulation is highlighted in the text.

The authors should better analyze this difference, also documenting membrane potential as a function of time in figures.

Reply: Unfortunately we did not measure the resting membrane potential continuously during ZD7288 wash-in, but Em was assessed at a few time points between other experimental protocols (such as between experiments delivering membrane potential steps at −70 mV holding potential). Therefore, we could only report the Em at specific time points (at baseline and 5 min following ZD7288 wash-in).

6. The action potential phenotype of the recorded human interneurons needs to be better described. Most importantly, both f-I curves and maximal action potential frequency must be given. The authors repeatedly refer to the recorded cells is fast spiking, but the most important criterion (high action potential frequency) is never demonstrated.

Reply: We have provided all electrophysiological parameters for each cell, including action potential kinetics and the firing frequency adaptation index, in Supplementary table 1. Although maximal firing frequency was not measured (in fact, intensive stimulation was intentionally avoided to prevent certain physiological changes), frequency adaptation was measured during robust (if not maximal) firing as suggested by the Petilla conference review on interneuron firing type characterization (“Petilla Interneuron Nomenclature G, Ascoli GA et al…”). Along with fast spike kinetics, frequency adaptation is a good functional marker for both rodent and human fast spiking pv interneurons (Szegedi et al. 2016, 2018, and 2020). We regret that not all cells in the study were successfully tested for pv immunoreaction. Nonetheless, we can confirm that if we restricted the species comparisons in Figure 1 to cells that were immunohistochemically confirmed to be pv-positive, the results show statistically significant species difference.

7. The nucleated patch recordings are nice, representing the strongest evidence for the hypothesis that HCN channels are enriched somatically. However, the data shown are incomplete. First, the number of experiments is unclear.

Reply: We have included comprehensive outside-out patch data for human fast-spiking and some mouse pv cells with statistical analysis. The numbers of cells are clearly stated in the text and relevant figure legend (and details for their fast-spiking phenotype are listed in Supplementary table 1).

Second, a quantitative  comparison between mouse and human is missing.

Reply: In the revised manuscript, we have included quantitative comparisons of somatic membrane outside-out patch data between species along with relevant statistical analysis.

Finally, analyzing the properties of nucleated patches in current-clamp conditions is unusual. As the input resistance is ~0.5 GOhm, the seal resistance may contribute to the voltage responses. Voltage-clamp experiments would be preferable, directly revealing the HCN conductance density.

Reply: We recognized that an extremely high input resistance increases the error of membrane potential reading. However, in most experiments, the increase in input resistance was only moderate. In addition, our outside-out-patch and whole-cell recordings in current clamp were obtained via full bridge balance compensation, which was initially specifically adjusted in whole-cell mode and was then adjusted in the outside-out mode, which minimizes voltage reading errors by the current steps.

Minor points:

Page 2, center: "that regulate within neural networks" - unclear.

Reply: We apologize for this error. It has been corrected as follows: *“…outputs that regulate neural networks*.”

Page 5, top: How many nucleated patch recordings were performed?

Reply: The number of nucleated patch experiments is provided in the revised Results and the legend of new Figure 4.

Page 6, top: "depolarizing effect" - shouldn't it be "hyperpolarizing"?

Reply: This has been corrected. Thank you.

Page 6, center: "in contrast … failed to induce" - in rats slices, ZD 7288 induces a consistent hyperpolarization (Aponte et al., 2006) - see major points above.

Reply: The section containing this statement has been rewritten.

Page 7, bottom: Classical studies suggested the presence of HCN1 immunoreactivity in apical dendrites of neocortical pyramidal neurons (Lörincz et al., 2002, Nature Neuroscience). Clarification is needed here.

Reply: The section containing this statement has been rewritten.

Page 9, bottom: "Excitatory currents simulating synaptic excitatory postsynaptic currents EPSCs …" - If this is a dynamic clamp experiment, taking into account changes in driving force, the statement is not quite correct.

Reply: The section containing this statement has been rewritten as follows (also highlighted in the manuscript text): “*Figure 5B1 illustrates how conductance was applied to dynamic clamp input to generate excitatory currents in cell soma mimicking the glutamatergic EPSCs reported in human fast-spiking cell soma by whole-cell recordings that generate spikes*”

Page 10, top: "for a mean" - unclear. "Only showed" - is it "only one showed"?

Reply: The sentence has been revised to *“,… only one of four mouse cells demonstrated a significant increase in delay following ZD7288 treatment.*”

Page 10, center: It is unclear why inwardly rectifying K+ channels were included in the model of the fast-spiking neuron. If available, functional evidence should be cited.

Reply: We have explained the inclusion of Kir in the revised Methods and Results sections as follows: “*The Kir conductance replicated the decreased input resistance observed during the application of hyperpolarizing currents from −20 to−100 pA in the presence of ZD7288 (Figs. 6A2 and B2).”*

Page 12, top: "compared rodent basket cells" should read "compared to rodent basket cells".

Reply: Thank you. This phrase has been corrected.

Page 12, bottom: "energy expenditure" - it is not easy to see why an Ih current should be more energy-efficient than a leakage potassium current. This argument should be better worked out.

Reply: We agree with the reviewer. This sentence has been removed.

Page 13, center: "and possible those" should read "and possibly those". It should be possible to figure out all these small annoying mistakes before submission.

Reply: Thank you for your attention to detail. We have corrected this error.

Page 14, top: That the change in the membrane time constant should affect the spike lag is not straightforward. The membrane time constant will primarily determine the decay of the EPSP, whereas the time to peak will be primarily determined by the time course of the AMPA receptor-mediated conductance.

Reply: We have revised our discussion of the effects of τ on EPSP–spike coupling.

Page 14, center: That perisomatic leak conductance enhances the fast signaling properties of fast-spiking parvalbumin-positive interneurons has been previously suggested by Noerenberg et al., 2010, PNAS. This aspect should be better discussed in the manuscript.

Reply: We thank the reviewer for pointing this out. We have cited this important article in our manuscript Discussion (also highlighted in text): *“Thus, in human fast-spiking neurons, the perisomatic HCN expression appears to act as a mechanism ensuring rapid and reliable EPSP–spike coupling [82]*.”

Page 14, center: Previous work showed that the kinetics of disynaptic inhibition may not only be important for processing speed, but may also be critical to implement novel computations, such as pattern separation (de Almeida et al., 2009, J. Neuroscience). This aspect works in favor of the author's argument and may be added.

Reply: We thank the Reviewer for this reference.

Page 14, bottom: "HCN channel activity can be rapidly up- or downmodulated through intracellular calcium-dependent processes" - this is misleading - the classical intracellular second messenger regulating HCN channels is cAMP.

Reply: This has been rewritten as follows: *“In some neuron types, HCN channel activity can be rapidly up- or downregulated by second messenger cascades initiated by neuronal excitation and activity.”*

Page 19, top: Unspecific wording like "in general" should be removed.

Reply: Most instances of “in general” have been removed.

Page 23, bottom: What is "lg / kg" ?

Reply: We apologize for this typo. It has been revised to “μg/kg.”

Page 24, top: It is unclear why the Cre line should express tdTomato. Was there a crossing with a reporter line (Ai9 or Ai14)?

Reply: We apologize. This is explained more clearly in the revised text (Methods and Results) and figure legends.

Page 24, bottom: "effect of access resistance… was calculated and corrected during data analysis" - if the authors want to achieve an accurate correction, they need to perform series resistance compensation during the experiment at the amplifier level.

Reply: In some experiments, bridge balance compensation was performed post hoc during data analysis, which is acceptable because the microelectrode time constant is extemely short and the access resistance-derived component is clearly distinguishable in the early voltage response to a square-pulse current step.

Page 25, center: If recordings are made at physiological temperature, as done here, 3 ms decay time constant is too slow for AMPA receptor-mediated EPSCs in parvalbumin-expressing interneurons.

Reply: In human pv neurons, large excitatory monosynaptic currents, which are measured in voltage clamp mode at the soma, decay with a time constant of 1.2–3 ms (e.g. Szegedi et al. 2016, cited in the manuscript). These currents are multivesicular (e.g., Molnar et al. 2016 cited here).

Page 25, bottom: "in addition to HCN channels … h current" - unclear - isn't the h current the correlate of the HCN channels?

Reply: We have used HCN channels, HCN current, and HCN conductance (gHCN) throughout the revised manuscript.

Page 26: Equations should be numbered for clarity.

Reply: Equations have been numbered from 1 to 7.

Page 26 / 27: Versions of programs used should be specified.

Reply: Software versions have been provided in the revised Methods section.

Page 28, top: Numerical aperture of objective should be mentioned.

Reply: The NA of the objective used for confocal and STORM studies has been indicated (NA = 1.49).

Page 28, center: As parvalbumin immunoreactivity will be washed out during patch-clamp measurements, recording times and access resistance should be quantified in the methods section.

Reply: We have added the following statement (also highlighted in Methods): “*All cells experiments were performed within 30 min from entering to whole cell mode.*”

Legend figure 1: "hydrocephalus" is misspelled.

Reply: This error has been corrected.

Legends throughout: The authors should make clear at which time point after ZD application measurements were taken.

Reply: The time of measurements after ZD is now clearly indicated in the revised text and figure legends. Recordings were started at least 5 min after drug wash-in.

Throughout the paper, the authors refer to patches as outside-out patches, macropatches, or nucleated patches. A more consistent nomenclature should be chosen (previous papers widely called them nucleated patches).

Reply: We have consistently referred to the recordings as “outside-out patch.”
